# Diffusion Weighted Imaging in Neuro-Oncology: Diagnosis, Post-Treatment Changes, and Advanced Sequences—An Updated Review

**DOI:** 10.3390/cancers15030618

**Published:** 2023-01-19

**Authors:** Andrea Romano, Serena Palizzi, Allegra Romano, Giulia Moltoni, Alberto Di Napoli, Francesca Maccioni, Alessandro Bozzao

**Affiliations:** 1 NESMOS Department, U.O.C. Neuroradiology, “Sant’Andrea” University Hospital, 00189 Rome, Italy; 2 IRCCS Fondazione Santa Lucia, 00179 Rome, Italy; 3 Department of Radiology, Sapienza University of Rome, Viale Regina Elena 324, 00161 Rome, Italy

**Keywords:** diffusion-weighted imaging, magnetic resonance imaging, tumors

## Abstract

**Simple Summary:**

Differential diagnosis among primary and secondary central nervous system tumors, the assessment of tumoral therapy response, the correlation between imaging and prognosis, and biomolecular findings remain the main diagnostic challenges in the neuro-oncology imaging field. In the literature, novel, sophisticated MRI sequences and applications of artificial intelligence are frequently researched with this goal in mind; anyway, some of these tools are still not applicable in clinical practice. On the contrary, Diffusion Weighted Imaging (DWI) is a sequence commonly used in everyday clinical practice that can give information in terms of tumor grading, differential diagnosis, molecular profile, and response to therapy.

**Abstract:**

DWI is an imaging technique commonly used for the assessment of acute ischemia, inflammatory disorders, and CNS neoplasia. It has several benefits since it is a quick, easily replicable sequence that is widely used on many standard scanners. In addition to its normal clinical purpose, DWI offers crucial functional and physiological information regarding brain neoplasia and the surrounding milieu. A narrative review of the literature was conducted based on the PubMed database with the purpose of investigating the potential role of DWI in the neuro-oncology field. A total of 179 articles were included in the study.

## 1. Introduction

Diffusion-weighted imaging (DWI) is a magnetic resonance imaging (MRI) sequence commonly used in neuroradiology for the assessment of acute ischemia, inflammatory diseases, and central nervous system (CNS) neoplasia [1,2,3].

It has several advantages, as it is a fast, easily reproducible, and extensively studied sequence in neuro-oncology, and it is widely available on many standard scanners, including those in non-academic centers [4,5].

In addition to being a simple instrument for everyday use, DWI can also offer functional and ultrastructural data, particularly when discussing tumor cellularity and the microenvironment through the measurement of water mobility. In fact, it yields an imaging biomarker for pathological tissue changes like cellularity increase or anomalies in the extracellular space by the assessment of water mobility [6,7,8].

Moreover, advanced diffusion-related sequences such as Diffusion Tensor Imaging (DTI) and Diffusion Kurtosis Imaging (DKI) are even playing a more important role than DWI in the neuro-oncology field.

Finally, multi-shell diffusion MRI allows for characterizing the water diffusion signal behavior by analyzing the data using multi-compartment diffusion models such as the Neurite Orientation Dispersion and Density Imaging (NODDI) model. Thus, it can provide a more specific characterization of brain tissue microstructures than conventional single-shell diffusion tensor imaging.

## 2. Gliomas and Cellularity

**Figure 1:**
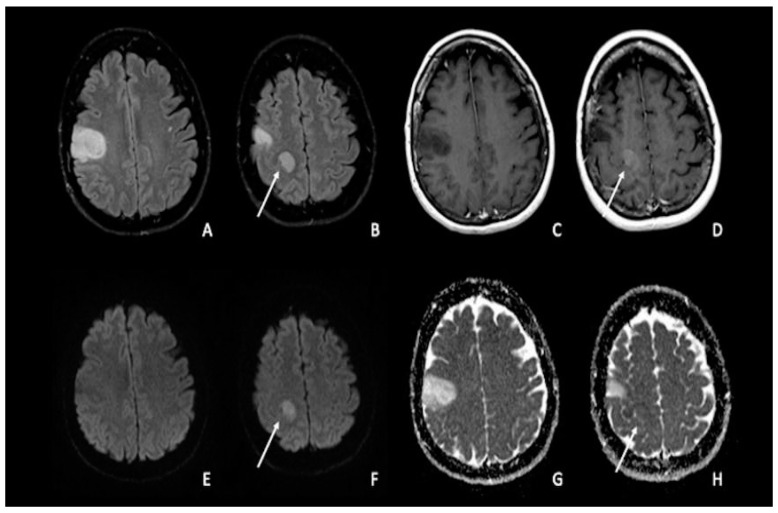
Double site of glioma infiltration in the right Rolandic frontal region: (**A**,**B**) Both lesions present similar hyperintensity on FLAIR images. The most cranial lesion (arrow) showed slight post-contrastographic enhancement (**D**) and restricted water diffusion on DWI (**F**), characterized by slightly low values on ADC map (**H**); this condition could be linked to an anaplastic aspect of the tumor, with restricted diffusion explained by an increase in tumor cellularity. The most caudal lesion did not show contrast enhancement, with a marked hypointense signal on post-contrastographic T1-weighted image (**C**); the same lesion presented no restricted diffusion with increased ADC values (**E**–**G**). FLAIR = fluid-attenuated inversion recovery; DWI = diffusion-weighted imaging; ADC: apparent diffusion coefficient.

Brain tumors may show various degrees of diffusion changes related to tumor cellularity and the nucleus-cytoplasmic ratio [9]. Water diffusion restriction secondary to tumor high-cellularity results in low Apparent Diffusion Coefficient (ADC) values, which are useful for differentiating tumor type and grade [6,10,11]. The minimal ADC value has been proposed as one of the various parameters to use as a predictive tool in patients with malignant supratentorial astrocytomas [12,13,14,15,16,17,18]. For instance, Moon et al. [12], Yamasaki et al. [13], and Murakami et al. [14] showed an inverse correlation between the mean ADC value and tumor grade in astrocytic tumors. Generally, higher-grade tumors typically have lower ADC values (Figure 1A–H). Due to their variable cellularity and grade, astrocytomas exhibit heterogeneous diffusion signals, with most cellular areas generally exhibiting restricted diffusion [9]. For instance, the degree of ADC hypointensity will often be higher in lymphoma than in glioma or metastases, reflecting the high cellular density in this neoplasm. The drop in ADC values for non-necrotic high-grade gliomas and metastases will be higher than for low-grade malignancies. High-grade tumor-associated edema reduces ADC sensitivity by raising the average ADC intensity [6,7]. ADC values alone or in combination with other MRI parameters, such as relative cerebral blood volume (rCBV), derived by dynamic susceptibility contrast-enhanced MR-perfusion (DSC), can accurately grade gliomas [19]. Indeed, Wang et al. recently reported a high accuracy of DWI/ADC to distinguish low-grade gliomas (grades I and II) from high-grade ones (grades III and IV) with an area under the curve (AUC) of 0.91; therefore, with this value being very high it reflects an excellent diagnostic performance of DWI in this field [9,20].

In addition to standard DWI acquisition, more advanced models of quantitative DWI, such as DTI and DKI, have been created. These advanced sequences try to go beyond the theory that water diffusion occurs without boundaries via a uniform Gaussian distribution; indeed, it also depends on the configuration of intracellular organelles, cell membranes, and water compartments in cerebral tissues [6,21,22]. For instance, thanks to DKI, it is possible to quantify the deviation from a Gaussian distribution to produce a more accurate model [6,23,24]. The same result was reached by Abdalla et al. in a more recent expanded and updated meta-analysis, which found that DKI offers good diagnostic accuracy in differentiating high-grade from low-grade gliomas [6,25].

Finally, intravoxel incoherent motion (IVIM) and neurite orientation and dispersion imaging (NODDI), which make use of multiband imaging, are two promising advanced DTI approaches [6,26]. NODDI, in particular, measures the microstructure of dendrites and axons, revealing neuronal alterations, whereas IVIM may estimate tissue diffusivity and microcapillary perfusion [6].

## 3. Gliomas and Molecular Biology

**Figure 2:**
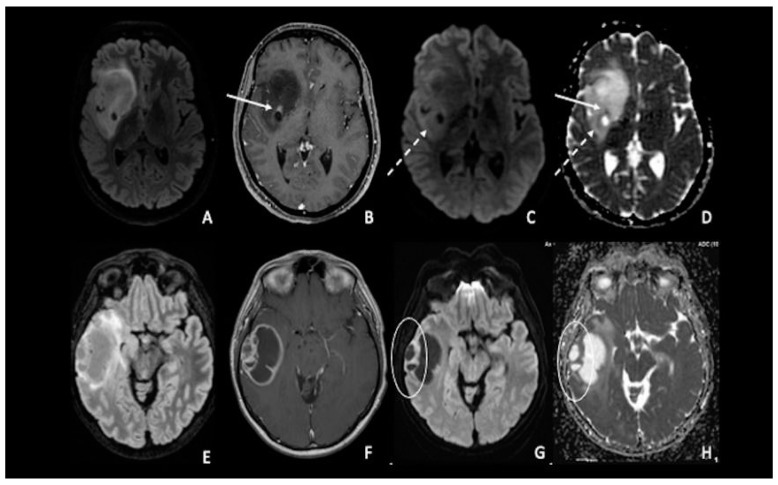
(**A**–**D**) IDH-1 mutated glioma in the right Sylvian region. The lesion is characterized by a heterogenous signal on FLAIR (**A**), with a small area of nodular enhancement on post-contrastographic T1 weighted images (arrow) (**B**); The site of pathological enhancement was in close proximity to an area of cystic-necrotic degeneration (dotted arrow) (**C**,**D**) and showed restricted diffusion on DWI confirmed by the ADC map (arrow); these findings were consistent with an anaplastic behavior of the tumor. (**E**–**H**) Right temporal lobe glioblastoma. The lesion was predominantly cystic and had a vivid peripherical post-contrastographic enhancement (**F**) with a solid component on the lateral side of the tumor; this region presented restricted diffusion on DWI confirmed by the ADC map (circle) (**G**,**H**), related to an increase in tumor cellularity. FLAIR = fluid-attenuated inversion recovery; DWI = diffusion-weighted imaging; ADC = apparent diffusion coefficient.

When considering the glial line neoplasm, the 2016 World Health Organization (WHO) classification system has defined various groups of diffuse low-grade gliomas (LGGs) considering the isocitrate dehydrogenase (IDH) mutation and 1p/19q codeletion status [27,28]. The WHO classification of 2021 increased the importance of the molecular profile of brain tumors by categorizing adult-type diffuse glioma into three groups: IDH mutant with 1p/19q codeletion (Oligodendroglioma/IDH-mut codeleted LGGs), IDH mutant without codeletion (Astrocytoma/IDH-mut non-codeleted), and IDH wild type (Glioblastoma) [29]. The IDH gene plays a crucial role in metabolism, cellularity, and angiogenesis [30]. Recent research has shown that IDH mutant gliomas exhibit significantly greater survival and chemosensitivity than IDH wild-type glioblastomas [31,32]. When the IDH gene family is mutated, an oncometabolite called 2-hydroxyglutarate is produced, which inhibits tumor cell proliferation more than the wild type [33,34]. When compared to astrocytomas, oligodendrogliomas have a superior clinical outcome and treatment response [29,31,35,36,37]. As IDH mutant inhibitors become commercially available and might even be employed as neoadjuvant therapy, imaging biomarkers of IDH mutation would be a useful adjunct tool for clinical decision-making [38]. As a result, numerous research studies [33,39,40,41,42,43,44,45,46,47,48,49,50,51,52,53,54,55,56,57,58,59,60,61,62,63,64,65] investigated the imaging properties and/or high diagnostic performance of various MRI sequences for the prediction of IDH mutations in gliomas. According to several studies [41,48,56,60,62], IDH mutant glioma consistently displays higher mean ADC values on DWI than IDH wild-type glioblastoma [34]. It is still unclear how IDH-mutant and IDH wild-type gliomas differ from one another in terms of the ADC values; however, it could possibly be primarily related to tumor cellularity but also to the presence of cystic components, areas of necrosis, and interstitial water content [17,18,66]. While most IDH wild-type gliomas commonly present high-grade features such as necrosis and lower ADC mean values in solid sections, perhaps indicating more cellularity, most IDH-mutant gliomas show higher ADC mean values and MR imaging features consistent with a lower-grade nature [37] (Figure 2A–H). When considering oligodendrogliomas and the relationship between the 1p/19q codeletion status and ADC levels, the literature has shown contradictory findings [56,67,68,69]. According to Jenkinson et al., IDH-mut non-codeleted LGGs had much higher ADC values than IDH-mut-codeleted LGGs. Compared to IDH-mut non-codelated LGGs, the IDH-mut codeleted group may have fewer edematous areas and more cellulated areas, which could account for the lower ADC values [29,68]. Other diffusion-based sequences, such as DTI, DKI, and NODDI, have been successfully tested in predicting IDH status in gliomas, both with and without the use of artificial intelligence [70,71], with similar results between DTI and more advanced multi-shell methods [71,72]. Interestingly, mean diffusivity (MD) measures were increased in tumors not usually associated with high cellularity, probably reflecting changes in the extracellular volume that play a role in the diffusion signal [71]. Epidermal Growth Factor (EGFR) amplification is a molecular biomarker that could allow glioblastoma IDH wild-type designation even in tumors that appear histologically lower grade [29]. Hence, its prediction through MRI could be of therapeutic and prognostic use. A recent pilot study found that EGFR-amplified tumors showed lower mean ADC values compared to EGFR-non-amplified tumors [73]. Future research could focus on other important molecular information in gliomas, such as CDKN2A/B deletion or combined whole chromosome 7 gain and whole chromosome 10 loss (+7/−10) mutations.

## 4. Lymphomas

**Figure 3:**
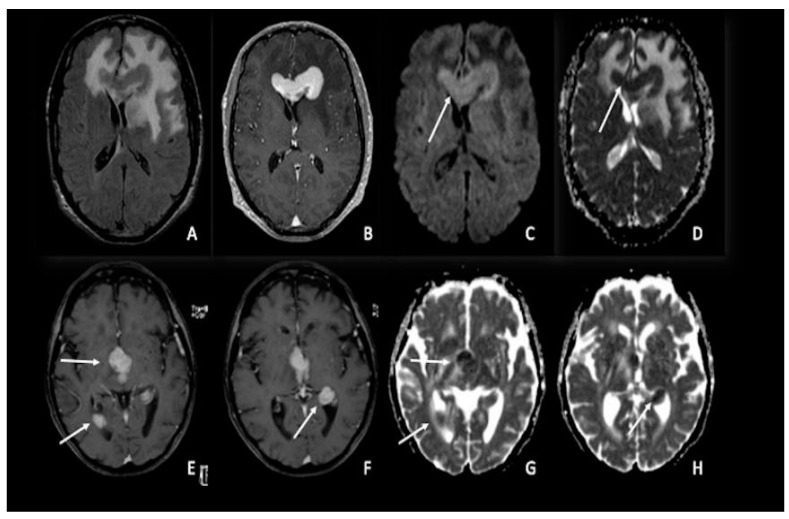
Two different cases of primitive cerebral lymphoma: the upper row showed a lymphoma in the pericallosal region (**A**–**D**) with a marked uniform enhancement on post-contrastographic T1 weighted image (**B**) and significant edema in the adjacent brain (**A**). The lower row showed a periventricular multicentric lymphoma (**E**–**H**) with multiple nodules of marked enhancement on post-contrast T1 weighted images (arrows) (**E**,**F**). Both cases presented restricted water diffusion with hyperintensity on DWI (arrow in **C**) and hypointense areas on ADC (arrows respectively in (**D**,**G**,**H**)), related to increasing tumor cellularity, as occurs typically in lymphomas. DWI = diffusion-weighted imaging; ADC = apparent diffusion coefficient.

One of the uses of DWI on CNS neoplasia imaging is the differential diagnosis between glioblastoma and primary CNS lymphoma (PCNSL), two entities that may appear similar on conventional imaging when considering a patient presenting with an enhancing brain mass but with a completely different pathogenesis, origin, and treatment [74,75]. PCNSLs are highly cellular tumors with relatively little extracellular space, which limits the diffusivity of free water. As a result, compared to HGGs and metastases, PCNSLs have been found to have much lower ADC values (Figure 3A–H). Similar to ADC, PCNSLs have been found to have lower FA values than high-grade gliomas [4,76,77,78,79,80,81,82,83,84,85].

## 5. Medulloblastomas

**Figure 4:**
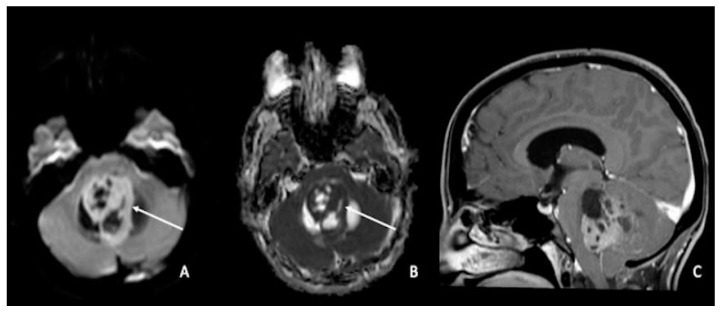
Medulloblastoma in a 30-year-old: (**A**,**C**) lesion of the posterior fossa with growth in the median line, close to the IV ventricle, characterized by inhomogeneous enhancement (arrow in (**A**)), mass effect, and (**B**) low ADC values (arrow). Using ADC values and a specific cut-off, it could be possible to distinguish medulloblastoma from ependymoma or pilocytic glioma. ADC = apparent diffusion coefficient.

Medulloblastomas are highly cellular tumors; consequently, they present a substantial reduction in water molecule movement, resulting in a high diffusivity restriction on DWI and ADC images [86,87,88,89,90,91,92,93,94,95,96,97,98,99,100,101,102,103]. This finding helps in the differential diagnosis of other tumors typically located in the posterior fossa, such as ependymomas and pilocytic astrocytomas. Indeed, even if there is still overlap between these tumors [94,96,97,103,104,105,106], a high diffusion restriction is more suggestive of medulloblastomas than ependymomas or pilocytic astrocytomas [86,103,107] (Figure 4A–C). An ADC cut-off between 700 and 900 mm^2^/s has been proposed by the literature to distinguish medulloblastomas from pilocytic astrocytomas [106,108,109], whereas using a minimum ADC cut-off value of 660 mm^2^/s seems to allow for a good distinction with ependymomas [110]. Moreover, according to the literature, the ratio of ADC within the tumor compared to the grey matter ranges between 0.70 and 0.88 for the solid component [97,108,111] and 0.97 and 1.28 for the entire tumor [108,112,113,114]. Medulloblastomas, ependymomas, and pilocytic astrocytomas share the same fractional anisotropy [115,116,117]. As measured by lower ADC values, medulloblastomas frequently have lower rates of microscopic water diffusion than other common posterior fossa tumors in children [96]. This trait is most likely brought on by the frequent presence of cells with a high nuclear-to-cytoplasmic ratio and a high degree of cellularity in medulloblastomas, which results in additional membrane barriers impeding microscopic water diffusion [118]. Another much rarer tumor of the posterior fossa, typically affecting children, is the atypical teratoid/rhabdoid tumor (ATRT), which histologically resembles medulloblastoma [119] and exhibits similar DWI characteristics to medulloblastoma [86,111]. Finally, the distinction between medulloblastomas and glioblastomas cannot be made with DWI [86].

According to Ahmed et al.’s study, the ADC ratio (ADC of the tumor divided by the ADC of the corresponding contralateral normal white matter) was unable to distinguish between medulloblastoma and ATRT. This could be explained by the fact that medulloblastoma and ATRT both have high grades in accordance with the WHO grading system, which indicates high tumoral cellularity and leads to a low ADC ratio [120].

## 6. Meningiomas and Vestibular Schwannomas

More than 30% of all brain tumors are meningiomas, which are the most frequent benign intracranial tumors [1]. Meningiomas are classified with three malignancy grades (WHO grades 1–3) in the 2016 World Health Organization (WHO) classification of central nervous system tumors [2] and are considered a single tumor type with 15 subtypes in the 2021 WHO classification. The more aggressive meningiomas are clinically characterized by more morbidity and mortality and have a higher chance of recurrence [3,4]. Tumor grading has a significant role in meningioma therapy planning. Adjuvant radiation is advised for high-grade meningiomas, while surgical excision is seen to be appropriate for low-grade meningiomas [5,6]. Therefore, accurate preoperative tumor grade prediction for meningiomas is essential in clinical practice.

**Figure 5:**
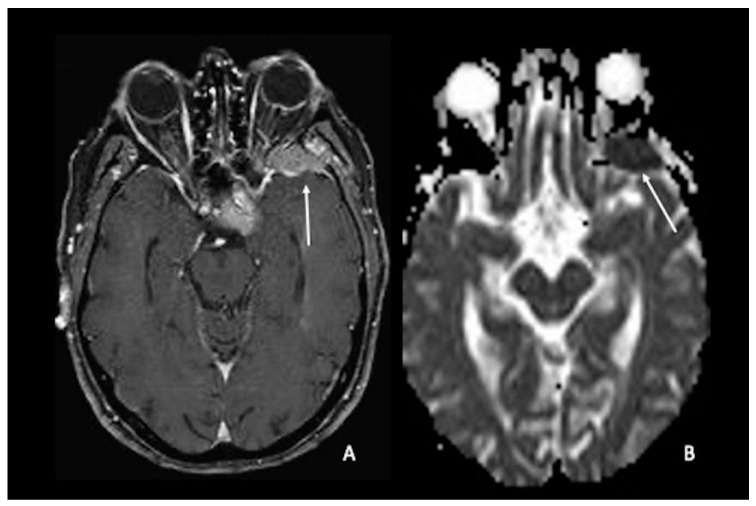
Aggressive meningioma: (**A**) atypical or malignant meningiomas (arrow) could have (**B**) lower ADC values than normal brain parenchyma (arrow). ADC = apparent diffusion coefficient.

Numerous studies have described the correlation between ADC values and the grading of meningiomas, with debated results. The ability of DWI to classify cellular malignancies has allowed it to characterize various meningioma subtypes. Except for heavily calcified or psammomatous meningiomas, atypical or malignant meningiomas have lower ADC and higher FA values than normal brain parenchyma [121,122,123] (Figure 5A,B). According to the WHO 2016 classification, meningiomas are graded based on the mitotic number and the invasive characteristics. The Ki-67 index is a crucial indicator of cellular proliferation, and research has shown a relationship between the Ki-67 index and the grading of meningiomas [124]. In addition, meningiomas with a higher Ki-67 index have a worse prognosis and a higher probability of tumor recurrence [125]. Numerous cancers’ microstructural cellularity was demonstrated to be reflected by ADC values [126]. In fact, some authors claimed that ADC values could distinguish between low-grade and high-grade meningiomas by correlating inversely with the Ki-67 proliferation index [127]. This paper showed that mean ADC levels in high-grade meningiomas are substantially lower than in low-grade meningiomas. As a result, ADC may be a practical and non-invasive method for identifying low-grade and high-grade aggressive meningioma as well as for planning meningioma treatments, such as the degree of tumor resection, the application of adjuvant radiation, and the intervals for MRI follow-up [127]. The validation of an ADC threshold, however, was not observed in an older meta-analysis of 17 trials that were conducted to differentiate between benign and high-grade meningiomas [128]. Diffusion MRI was also used to predict meningioma consistency, with debated results. Although Hoover et al. and Watanabe et al. did not find any association with tumor consistency [129,130], Yogi et al. discovered that hard meningiomas had considerably lower minimum ADC values than soft tumors [131]. Although they lacked histological confirmation, the authors concluded that tougher lesions are characterized by significant cellularity and fibrous material. Regarding the use of DTI, Kashimura et al. demonstrated that the FA values for hard meningiomas were significantly higher than those for soft meningiomas [132,133].

Vestibular schwannomas (VSs) are the most common tumors in the cerebellopontine angle [134]. The therapeutic options for VSs include observation, surgery, and radiation therapy [135], depending on different variables such as the size at initial diagnosis, the tumor growth rate on serial imaging, and patient symptoms. Radiation therapy is usually the better choice for small to medium-sized VSs, with a lower rate of facial nerve palsy and hearing loss in comparison with surgery [136].

Another important clinical-radiological issue is the early assessment of tumor response to therapy. Morphologic and volumetric changes represent useful information in response to treatment. Anyway, it seems that in the acute phase after radiotherapy (from week 2 to week 8), there is an acute response characterized by cell swelling and disruption of the cytoarchitecture due to radiation that leads to an increase in terms of tumor volume, making the volumetric measures useless in the early post-radiotherapy assessment [137]. On the other hand, it seems that DWI and DTI could provide functional information about the response after radiotherapy in VSs, making them useful tools [137].

Indeed, a cut-off of 800 × 10^−6^ mm^2^/s for minimum ADC values in VSs has been proposed to predict tumor response to radiotherapy with 90% accuracy, 77.8% sensitivity, and 100% specificity. All VSs from non-responders had ADC values greater than 800 × 10^−6^ mm^2^/s. No statistically significant correlation with tumor response is evident when comparing ADC values before and after radiotherapy, perhaps due to tissue damage and vasogenic edema development [138]. Other authors reported that DTI could detect functional changes in response to stereotactic radiosurgery that preceded morphological modifications. Starting from week 12 after treatment, significant changes in VSs were evident; a FA reduction and an ADC increase correspond, respectively, to disruption of the cytoarchitecture and necrosis [137].

## 7. Metastasis

**Figure 6:**
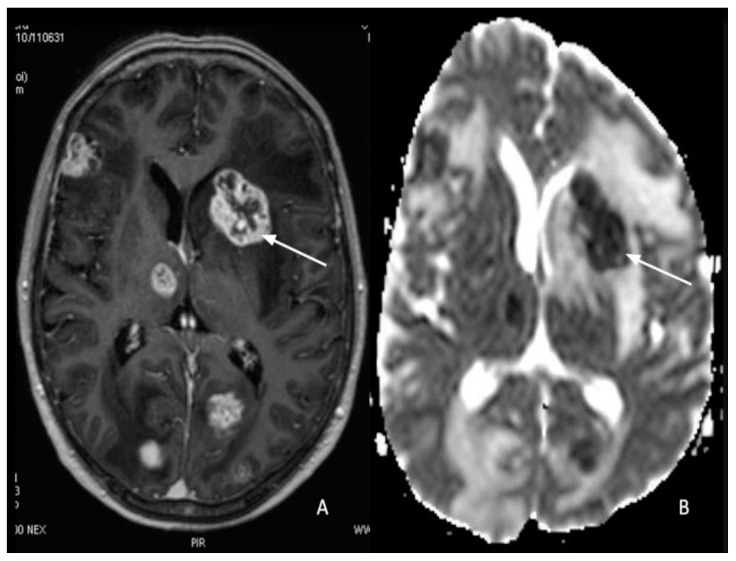
(**A**) Multiple enhancing metastatic lesions in a patient with lung cancer; (**B**) the reduced diffusion effect (arrow) is related to the high content of mucinous components.

Brain metastases are the most frequent central nervous system malignancies, often related to lung or breast cancer and less frequently related to cutaneous or intestinal tumors. Hematogenous spread is the most frequent way for cancer cells to move to the central nervous system. The spread of metastatic disease within the CNS follows different steps: first of all, there is the detachment of neoplastic cells from the primary tumor mass, then these cells enter the bloodstream with hematogenous disease dissemination to the metastatic site, and finally, there is the extravasation through the vascular wall and perivascular or brain parenchymal proliferation [139,140]. The ADC features of metastatic brain lesions and the distinction from primary tumors have only been the subject of a small number of studies, which may aid in distinguishing the origin of different brain metastases [141]. Restricted diffusion on DWI can be frequently found in intracerebral metastases, especially when the tumor is lung or breast cancer. The restriction of diffusion within a single brain metastasis may have been caused by an increase in the protein concentration in the form of highly viscous mucin [142] (Figure 6A,B). The pathology of metastases can be predicted by DWI signal intensity. On DWI sequences, adenocarcinomas (from the lung, ovary, and uterus) tend to be hypointense. Instead, hyperintense signals were seen in small- and large-cell neuroendocrine carcinomas [143]. Restricted diffusion was also observed in metastatic lesions of breast, colon, lung, and testis carcinomas, according to Duygulu et al. [142]. Studies have shown that small cell carcinomas (SCLC) tend to have low ADC values when compared to other tumors [144,145]. Other authors confirmed these results. Meyer et al. looked at 948 lesions from 159 patients, with malignant melanomas and lung and breast carcinomas being the most common types; due to lower values, ADC assessment allowed the differentiation of SCLC metastases from other tumors [146]. In another study, Zakaria et al. reported a negative correlation between cellularity and ADC values, with melanoma and SCLC having lower ADC values than metastases from other carcinomas (breast, ovarian, and colorectal malignancies) [147]. The genetic assessment of metastatic lesions may have an impact on the signal strength of lung cancer brain metastasis on DWI. Indeed, some authors find a significant correlation between ADC min and ADC ratio values and the presence or absence of EGFR mutations and their locations. In particular, low ADC values for lung adenocarcinoma-derived brain metastasis are linked to a high likelihood of an EGFR mutation, notably in exons 19 and 21 [148]. Multifocal, Dural-based enhancing lesions are the most frequent sign of metastatic disease to the dura.

**Figure 7:**
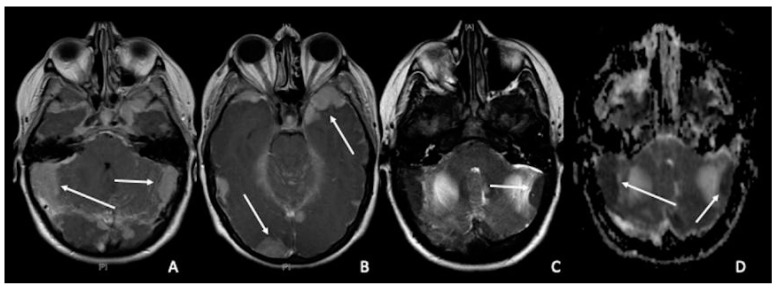
Multiple Chloromas (both supratentorial and infratentorial) in a patient with leukemia: (**A**,**B**) Pathologic extra-axial tissue enhancement after gadolinium (arrows) showed (**C**,**D**) restricted diffusion (arrows).

Pachymeningeal carcinomatosis in tumors outside the CNS is uncommon; however, it most frequently manifests as hematogenous dissemination [149]. Pachymeningeal carcinomatosis is frequently caused by secondary leukemia, lymphoma, and metastatic breast, lung, and prostate cancers and shows restricted diffusion [139]. (Figure 7A–D).

## 8. Gliomas vs. Metastasis

Diffusion imaging has also been used to discriminate glioblastoma from solitary brain metastases. Unfortunately, whereas tumoral ADC aids in tumor-type differentiation in some cases, it does not seem to be able to distinguish glioblastomas from metastases [13,150,151,152]. DWI can also be used to assess the ability of water molecules to move freely within non-enhancing peritumoral signal abnormalities (NEPSA) of CNS lesions. Neoplastic cells intermingled with areas of vasogenic edema within infiltrative NEPSA will create biological barriers that prevent the free passage of water molecules, resulting in lower ADC values than pure vasogenic edema [17]. Supporting this hypothesis, peritumoral ADC is lower in anaplastic astrocytoma and glioblastoma compared to metastases [151,152,153,154]. The non-infiltrative NEPSA vasogenic edema exhibits greater ADC values than the infiltrating NEPSA because there are no neoplastic cells to restrict the water molecules’ movement [17]. Beyond the region of T2 signal abnormalities, analysis of ADC in the normal-appearing white matter has also been conducted. Patients with glioblastoma and metastases had distinct minimum ADC levels in their normal-appearing white matter; however, this method, in clinical use, was constrained by its underwhelming sensitivity and specificity (both around 70%) [151,155]. In the post-surgical setting, restricted diffusion aids in the detection of cytotoxic edema [6]. Low ADC levels, which are also indicative of hypoxia (as observed in ischemic stroke or after surgery), suggest that variations in tumor hypoxia may affect tumor ADC values [156]. In the subacute setting, the associated parenchyma may enhance and be mistaken for tumor growth. Considering this, a baseline MRI is advised within 48 h of surgery [157]. Given the weaker ADC signal of the tumor, DWI can also be helpful for assessing treatment efficacy and distinguishing chemo-radiation-induced alterations from tumoral cells [6,158]. However, it is unknown what effect scarring and gliosis from chemotherapy or radiation have on ADC [129,156].

## 9. Post-Treatment Evaluation

Diffusion restriction inside a treated astrocytoma is likely associated with the recurrence and progression of the disease. Similarly, decreasing tumor burden correlates with increased diffusion during serial follow-up. ADC levels can also distinguish between post-radiation alterations (1.29–2.06, mean 1.570.35) and tumor growth (0.75–1.30, mean 1.140.18) [9,159]. A recent meta-analysis showed that DWI combined with MRI spectroscopy has very high sensitivity and specificity in differentiating recurrent glioma from radiation injury [160]. The current standard of care for patients with glioblastomas includes radiation (RT) and concurrent and adjuvant temozolomide (TMZ). The methylation state of the O-6-methylguanine-DNA methyltransferase (MGMT) gene promoter is a highly accurate predictor of the effectiveness of alchilant chemotherapy. As a molecular marker, MGMT is primarily used to assess how chemotherapy affects malignant gliomas and to identify new potential targets for tumor therapy [14,18,150,161]. Patients who showed methylation of the MGMT promoter had greater ADCmin values than those who did not, according to some authors, who also observed that the ADC ratio was much higher in the methylated group [15]. These authors assert that methylated MGMT tumors differ from unmethylated tumors in that they may be more varied or less cellular, less aggressive, and more amenable to therapy [15]. In previous research, DWI-ADC histogram values have been correlated with progression-free survival in glioblastoma patients. Specifically, broad and large histogram values predicted poor progression-free survival after surgery and radiochemotherapy [162]. MRI hyperintensity on T2 and fluid-attenuated inversion recovery (FLAIR) surrounding the enhancing portion of the tumor are common findings in higher-grade gliomas, which contain a combination of vasogenic edema and tumoral cells and are usually referred to as “non-contrast enhancing tumors” (NET) [163,164]. The analysis of this portion is essential in the evaluation of brain tumors as it may give valuable information on prognosis and tumor recurrence. Rathore et al. observed that on preoperative MRI, areas with increased rCBV and reduced ADC values were predictive of glioblastoma recurrence [165]. Moreover, a recent study found that ADC radiomic features on NET correlated with survival after >80% surgery and chemo/radiotherapy in younger patients (younger than 65 years old) [166].

## 10. Pituitary Adenoma

A pituitary adenoma is a common intracranial tumor that is preferably removed through transsphenoidal nasal surgery [167]. However, some characteristics hinder the possibility of this approach. Fibrous tumors, for instance, are difficult to remove by curettage and suction via this approach, usually requiring a second transcranial surgery [168]. Hence, assessing tumor consistency in the preoperative stage could be very important. Several studies investigated the correlation between DWI values and tumor consistency, with conflicting results [169,170,171]. Recent research evaluated macroadenomas’ collagen content (which is high in a harder tumor), finding that lower ADC values were associated with higher collagen content [172,173]. Another study successfully differentiated between functional and non-functional macroadenomas by analyzing ADC histogram features [174]. In addition, ADC histogram values (skewness and kurtosis) have been proven useful in predicting macroadenoma proliferative potential and recurrence after surgery [175].

## 11. Skull Lesions

Skull lesions are often incidental findings and are associated with nonspecific symptoms [176]. If CT is a helpful tool for detecting bony lysis, sclerosis, and calcification within the lesion or its margins, MRI is superior to CT in the demonstration of soft tissue components and extraosseous involvement [177].

CT and MRI studies for the morphological evaluation of lesions do not always offer adequate answers to allow a correct diagnostic-therapeutic procedure to identify which lesions need treatment and which need follow-up [178]. Undefined margins, sclerotic margins, or cortical flaws may not always be able to discriminate between benign and cancerous tissue that involves the skull [177].

Among MR techniques, diffusion plays an important role in defining cellularity and allows us to distinguish tissues that are much more aggressive compared to benign ones. ADC values have been found to be much greater in benign skull tumors than in malignant lesions, suggesting that this parameter may help to focus the differential diagnosis for ambiguous diseases involving the skull [177].

Some authors reported that a threshold ADC value of 0.966 ×10^−3^ mm^2^/s is useful to differentiate benign lesions from malignant lesions of the skull, with a good diagnostic value (AUC of 0.76) [179]. Nevertheless, some benign lesions with low ADC resulted in false positives, such as eosinophilic granulomas, epidermoid cysts, and the sclerotic variant of fibrous dysplasia. Other authors observed that the combination of conventional CT and MRI images with DWI could be applied to discriminate the skull base lesions where biopsies could be hazardous. The combination of CT, MRI, and DWI provided the best sensitivity for identifying benign from malignant skull lesions, whereas DWI alone provided the best specificity [177].

## 12. Conclusions

In conclusion, diffusion techniques, both conventional and advanced, have been proven useful in different applications in neuro-oncology imaging, ranging from differential diagnosis, histopathological composition, molecular profiling, prognosis, post-treatment response, and tumor recurrence. Being very common and easy to use in clinical practice, this sequence may be further implemented to help clinicians find new aspects of brain tumors and new therapeutic options.
